# The sulfur-related metabolic status of *Aspergillus fumigatus* during infection reveals cytosolic serine hydroxymethyltransferase as a promising antifungal target

**DOI:** 10.1080/21505594.2024.2449075

**Published:** 2025-01-17

**Authors:** Reem Alharthi, Monica Sueiro-Olivares, Isabelle Storer, Hajer Bin Shuraym, Jennifer Scott, Reem Al-Shidhani, Rachael Fortune-Grant, Elaine Bignell, Lydia Tabernero, Michael Bromley, Can Zhao, Jorge Amich

**Affiliations:** a Manchester Fungal Infection Group (MFIG), Division of Evolution, Infection, and Genomics, Faculty of Biology, Medicine and Health, University of Manchester, Manchester, UK; b Lydia Becker Institute for Immunology and Inflammation, School of Biological Sciences, Faculty of Biology Medicine and Health, Manchester Academic Health Science Centre, University of Manchester, Manchester, UK; c MRC Centre for Medical Mycology, University of Exeter, Exeter, UK; d Department of Life Sciences, Manchester Metropolitan University, Manchester, UK; e Mycology Reference Laboratory (Laboratorio deReferencia e Investigación en Micología LRIM), National Centre for Microbiology, Instituto de Salud Carlos III (ISCIII), Madrid, Spain; f CiberInfec ISCIII, CIBER en Enfermedades Infecciosas, Instituto de Salud Carlos III, Madrid, Spain

**Keywords:** *Aspergillus fumigatus*, sulfur metabolism, antifungal targets, *in vivo transcriptomics*, fungal virulence, hydroxymethyltransferase

## Abstract

Sulfur metabolism is an essential aspect of fungal physiology and pathogenicity. Fungal sulfur metabolism comprises anabolic and catabolic routes that are not well conserved in mammals, therefore is considered a promising source of prospective novel antifungal targets. To gain insight into *Aspergillus fumigatus* sulfur-related metabolism during infection, we used a NanoString custom nCounter-TagSet and compared the expression of 68 key metabolic genes in different murine models of invasive pulmonary aspergillosis, at 3 time-points, and under a variety of *in vitro* conditions. We identified a set of 15 genes that were consistently expressed at higher levels *in vivo* than *in vitro*, suggesting that they may be particularly relevant for intrapulmonary growth and thus constitute promising drug targets. Indeed, the role of 5 of the 15 genes has previously been empirically validated, supporting the likelihood that the remaining candidates are relevant. In addition, the analysis of gene expression dynamics at early (16 h), mid (24 h), and late (72 h) time-points uncovered potential disease initiation and progression factors. We further characterized one of the identified genes, encoding the cytosolic serine hydroxymethyltransferase ShmB, and demonstrated that it is an essential gene of *A. fumigatus*, also required for virulence in a murine model of established pulmonary infection. We further showed that the structure of the ligand-binding pocket of the fungal enzyme differs significantly from its human counterpart, suggesting that specific inhibitors can be designed. Therefore, *in vivo* transcriptomics is a powerful tool for identifying genes crucial for fungal pathogenicity that may encode promising antifungal target candidates.

## Introduction

*Aspergillus fumigatus* is a ubiquitous mold that normally lives in soil, feeding on decaying organic matter [1]. During its natural life cycle *A. fumigatus* produces thousands of spores, also known as conidia, that are disseminated in the air. Because of their high abundance and prevalence, it has been estimated that every human breathes several hundred of these conidia on a daily basis [2], and due to their small size (2–3 µm), they have the potential to penetrate deep into the respiratory tract and even reach the lung alveoli [3]. In immunocompetent individuals, this has no consequence as epithelial mucocilliarity and a proper immune response eliminate the spores very efficiently [4]. However, patients with an imbalanced immune response are susceptible to *A. fumigatus*, which can cause a wide range of diseases collectively termed as aspergilloses [5]. Chronic and invasive aspergilloses are life-threatening infections with high mortality rates, even in patients receiving antifungal treatment [6,7]. First-line therapy for aspergilloses is based on the use of azoles, the only class of antifungals that can be orally administered. Worryingly, the number of clinical *A. fumigatus* isolates resistant to triazole antifungals is increasing worldwide, which correlates with higher mortality rates [6,8,9]. Consequently, new agents to fight this fungal pathogen are urgently needed.

Metabolism is central to the virulence of fungal pathogens, therefore its targeting is considered a promising strategy for the development of novel antifungals [10,11]. Fungal sulfur metabolism is particularly interesting as it comprises routes and enzymes that are often not conserved in humans, thus it is considered a propitious source of novel antifungal targets [12]. Indeed, we have previously shown that proper regulation of sulfur metabolism is crucial for *A. fumigatus* infective capacity [13], demonstrated that biosynthesis of S-containing amino acids is required for virulence [14], and comprehensively characterized methionine synthase, an enzyme in the trans-sulfuration pathway, as a promising antifungal target [15]. Others have also shown that sulfur is involved in the biosynthesis of essential fungal metabolites [16] and have proposed sulfur assimilation and the trans-sulfuration pathway as sources of antifungal targets [17,18]. Here, we aimed to expand on this knowledge by using *in vivo* transcriptomics to better characterize the fungal sulfur metabolic status during infection of the murine lung, and to use the expression profile of genes to identify those that may be relevant for infection, proposing them as promising candidates for further investigations. However, detection and analysis of the fungal transcriptome during infection is a persistent challenge, as fungal RNA usually represents only between 0.05% and 0.5% of the total RNA isolated from infected organs [19]. To overcome this obstacle, we have used NanoString nCounter, a novel probe-based technology with excellent reproducibility, sensitivity, and specificity when compared to other transcriptomic methods such as microarray hybridization or RT-qPCR [20,21]. Its high sensitivity, being capable of detecting mRNA concentrations in the femtomolar range [22], makes this technology ideal for detecting and quantifying fungal RNA during infection. Furthermore, its incomparable specificity is fundamental for preventing crosstalk with the abundant host RNA. Using this technology, we have assessed the transcription profile of a selection of metabolic genes during infection of the murine lung in two models of immunosuppression and at three time-points post infection, and compared them with the expression profiles under a variety of *in vitro* conditions [23]. By these means, we have uncovered interesting aspects of the *in vivo* sulfur metabolic status and identified genes that can be proposed as relevant for growth in the tissues. We have further characterized a promising candidate, the cytosolic serine hydroxymethyltransferase (*shmB*) encoding gene, demonstrating that it is essential for *A. fumigatus* viability as well as for virulence and that it is promising to aim for the development of fungal-specific inhibitors against this target, which can serve to address the emerging global threat of antifungal resistance [24,25].

## Results

### Linear amplification of *Aspergillus fumigatus* mRNAs allows gene expression profiling of low-input *in vivo* samples

**Figure 1.**
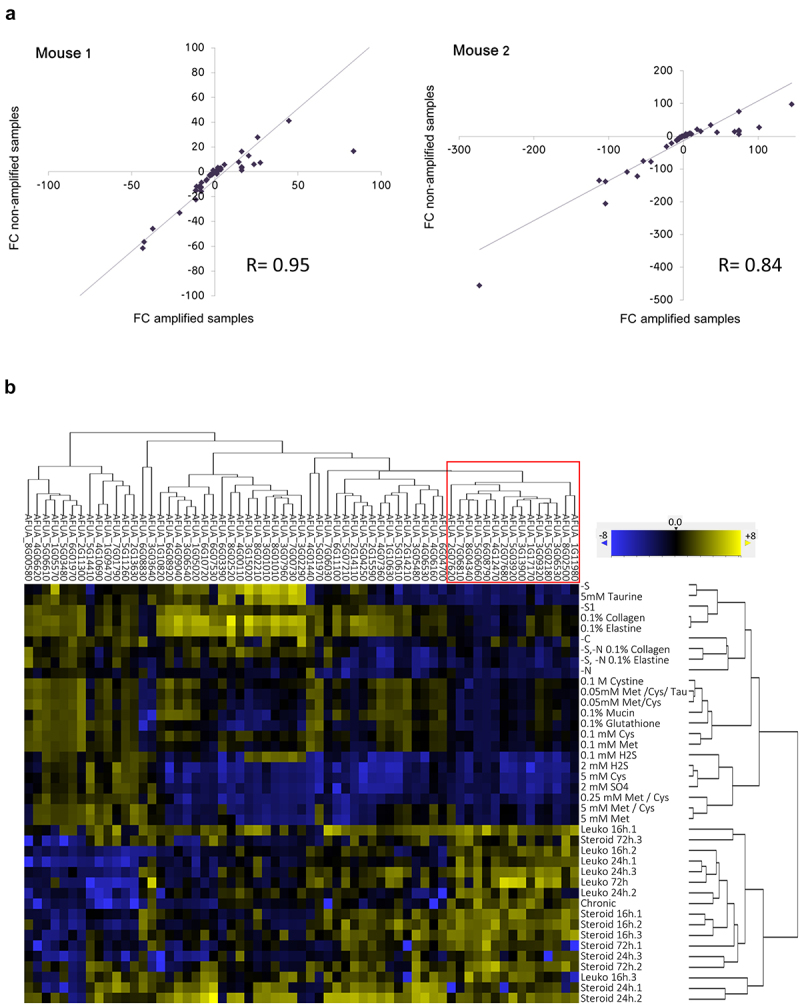
Identification of genes potentially relevant *for Aspergillus fumigatus* virulence using NanoString *in vivo* transcriptomics.

To determine the transcriptional status of genes related to sulfur metabolism during fungal infection of murine lungs, we developed a custom NanoString nCounter Elements TagSet comprising 68 metabolic genes (51 directly related to S-metabolism and 17 metabolic genes of interest indirectly related to S-metabolism) and 4 housekeeping controls (Table S1). We isolated RNA from *A. fumigatus* propagated under a variety of *in vitro* conditions (Table S2) and from the lungs of infected mice immunosuppressed with two different regimens, the leukopenic and the corticosteroid models (Table S2 and see Material and Methods). We isolated the lungs of infected mice at three different time points to investigate gene expression at early (16 h), medium (24 h) and late (72 h) time points of infection (three mice per time point, each constituting an experimental unit). We further assayed a chronic model of pulmonary aspergillosis [26] to determine whether this model provides a significantly different S-environment (Table S2). A preliminary experiment revealed that fungal RNA could not be reliably detected at 16 h and 24 h post infection, which was expected because the fungal burden at those early times is very low. To circumvent this problem, we applied the Nanostring Single-Cell Gene Expression protocol (see Materials and Methods) to perform linear amplification using Multiplexed Target Enrichment (MTE) primers. To verify that MTE enrichment does not affect the relative abundance of transcripts, we compared the fold changes between diluted-amplified and non-amplified (undiluted) of an *in vitro* sample (−S limiting conditions) versus *in vivo* samples (lungs extracted from two leukopenic mice 72 h after infection) (Figure 1a). This direct comparison demonstrated that the correlation of the fold changes detected with non-amplified and amplified samples was very high (R^2^ = 0.95 for mouse 1 and R^2^ = 0.84 for mouse 2). Therefore, MTE enrichment of low-input fungal RNA samples does not affect the relative abundance of transcripts and can be used to investigate gene expression in the mammalian lung at early time-points after infection.

a) Validation of the linear amplification performed with the Nanostring Single Cell Gene Expression protocol with Multiplexed Target Enrichment (MTE) primers. The fold changes between diluted-amplified and non-amplified (undiluted) *in vitro* (-S limiting conditions) and *in vivo* samples (lungs extracted from two leukopenic mice 72 h after infection) were compared. For both mice, there was a linear relationship between the FCs of non-amplified and amplified samples, demonstrating that amplification did not affect the relative abundance of transcripts. b) Hierarchical clustering of all samples formed two distinct branches, one of *in vitro* samples and one with the *in vivo* samples. There were 15 genes (red line) that were always expressed at higher levels in the *in vivo* samples, compared with all the *in vitro* samples.

### The fungal S-related metabolic status *in vivo* cannot be recapitulated *in vitro*, which can be leveraged to reveal genes potentially relevant for infection

To analyze the S-related fungal metabolic status *in vivo* and *in vitro*, we hybridized and read our custom NanoString codeset with isolated RNAs or amplified cDNAs (Table S2). The obtained raw data were subjected to quality control, which revealed that the samples obtained from two leukopenic mice at 72 h had not run properly; they were borderline for the recommended threshold of the overall number of counts (10,000) and did not reach the minimum values for the positive count reads (Fig. S1a and S1b). Consequently, these samples were excluded from all analyses. In addition, inspection of the normalized data (see Materials and Methods for details) revealed that five genes (AFUA_3G06492, AFUA_1G06940, AFUA_5G08600, AFUA_4G03950, and AFUA_6G00760) had very low counts (<20) in all samples. This could be due to the genes not being expressed at all or at very low levels in the tested conditions, or to the failure of the NanoString designed probes to detect these mRNAs. Accordingly, these genes were excluded from downstream analyses.

We first performed unsupervised hierarchical clustering of all analyzed samples (Figure 1b). Based on this stratification, the *in vivo* samples clearly clustered separately from the *in vitro* samples, indicating that the fungal S-related metabolic status during infection is significantly different from that during growth under diverse experimental conditions. This suggests that the lung provides a unique environment in terms of sulfur metabolism that is clearly distinct from defined culture media, despite our efforts to provide potentially relevant sulfur sources, and thus that we cannot predict the *in vivo* S-metabolic status in the laboratory. Notably, this also included the two S-depleted samples, which indicates that the mammalian lung is not sulfur-limiting. Based on this, we hypothesized that genes that are consistently more highly expressed *in vivo*, compared to the great variety of tested *in vitro* conditions, may be particularly relevant for the fungal capacity to grow in the lung tissues, and therefore for fungal virulence. We detected a cluster of 15 genes that were more highly expressed *in vivo* than *in vitro* (Figure 1b, red box, Table 1). Interestingly, we and others have already demonstrated the importance of at least five of those genes for *A. fumigatus* virulence, and a further six genes have already been proposed to be potentially relevant (Table 1 and see Discussion), supporting the rationale that the uncharacterized genes might be valid candidates for further analyses.

**Table 1.** Genes that consistently showed higher expression *in vivo* compared to any of the tested *in vitro* conditions.

GENE ID	DESCRIPTION	VIRULENCE
AFUA_1G11980	Glutamine amidotransferase (HisfF/H)	Suspected [27]
AFUA_8G02500	Glutathione S-transferase	Suspected [28]
AFUA_3G06530	ATP sulfurylase (sC)	Unknown
AFUA_5G02180	Cysteine synthase (CysB)	Proven [14]
AFUA_3G09320	Serine hydroxymethyltransferase (SmhB)	This work
AFUA_1G17170	Taurine dioxygenase	Unknown
AFUA_3G13900	Glutamate-cysteine ligase (Gcs1)	Suspected [28]
AFUA_5G03920	bZIP transcription factor (HapX)	Proven [29]
AFUA_2G07680	L-ornithine N5-oxygenase (SidA)	Proven [30]
AFUA_4G12470	bZIP transcription factor (CpcA)	Proven [31]
AFUA_6G08790	C6 transcription factor (PrnA)	Unknown
AFUA_5G06060	SCF-complex subunit (SkpA)	Suspected [32,33]
AFUA_8G04340	cystathionine gamma-lyase (MecB)	Proven [34]
AFUA_7G06810	L-amino acid oxidase (LaoA)	Suspected [35]
AFUA_2G07620	Cystathionine beta-synthase (mecA)	Suspected [34]

### Fungal sulfur related *in vivo* metabolism is influenced by immunosuppression and infection course

**Figure 2.**
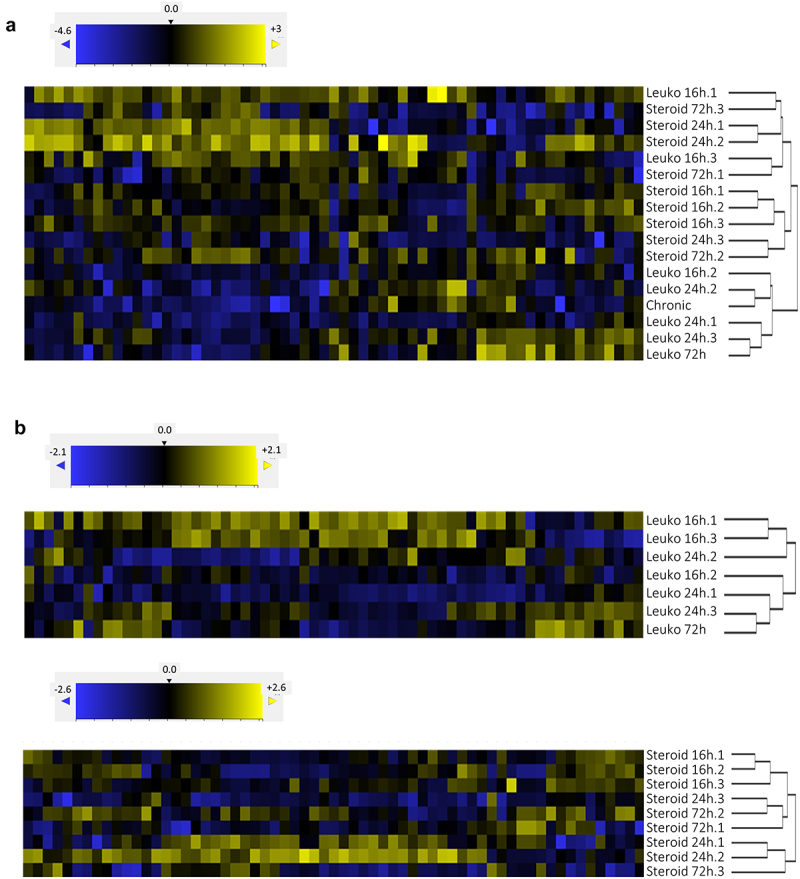
The fungal S-related sulfur metabolism *in vivo* is influenced by the model of suppression and the time of infection.

To investigate whether the fungal S-related metabolic status is influenced by the immunosuppression model, we performed hierarchical clustering of all the *in vivo* samples (Figure 2a). This clustering showed that the fungal S-related status is quite variable *in vivo* (note that the dynamic range of expression is much smaller than when comparing *in vitro* and *in vivo* samples in Figure 1b) and is partially dependent on the model of immunosuppression, as five out of the eight leukopenic mice aggregated in a separate cluster. Interestingly, the chronic model clustered in this separated branch, suggesting that the fungal S-related metabolic status in this type of infection is more similar to the status in the leukopenic model than in the steroid-induced model. Nevertheless, some samples from leukopenic mice interleaved with samples from steroid-treated mice, demonstrating that the model of immunosuppression is not the only factor that determines the fungal metabolic status.

a) Hierarchical clustering of all *in vivo* samples created two major branches, one almost exclusive for leukopenic samples, which only included the chronic model sample. The second major branch was divided again in two branches, one exclusive for steroid samples and the other mixed with leukopenic and steroid samples. Therefore, the model of suppression influences but is not the only factor that determines the fungal metabolic status. b) Separated hierarchical clustering of leukopenic or steroid samples partially aggregated by the time of infection. Therefore, the fungal S-related metabolism is influenced *in vivo* by the status of infection.

To investigate the extent to which the fungal S-related metabolic status is influenced by the course of infection, we performed hierarchical clustering of the samples from leukopenic and steroid-treated mice separately (Figure 2b). In both models of suppression there was a partial aggregation of samples according to the time point post infection, suggesting that the course of infection is mirrored by the S-related metabolic status of the fungal pathogen.

In conclusion, the immunosuppression model and the duration of infection appear to influence the status of fungal S-related metabolism. However, these variables alone or in combination cannot explain the variances among the samples, indicating the involvement of other factors. For instance, it is expected that the exact anatomical location of the foci of fungal growth would account for different microenvironments that in turn affect fungal S-related metabolism.

### Temporal dynamics of gene expression *in vivo* versus *in vitro* revealed the existence of potential disease initiation and progression factors

**Figure 3.**
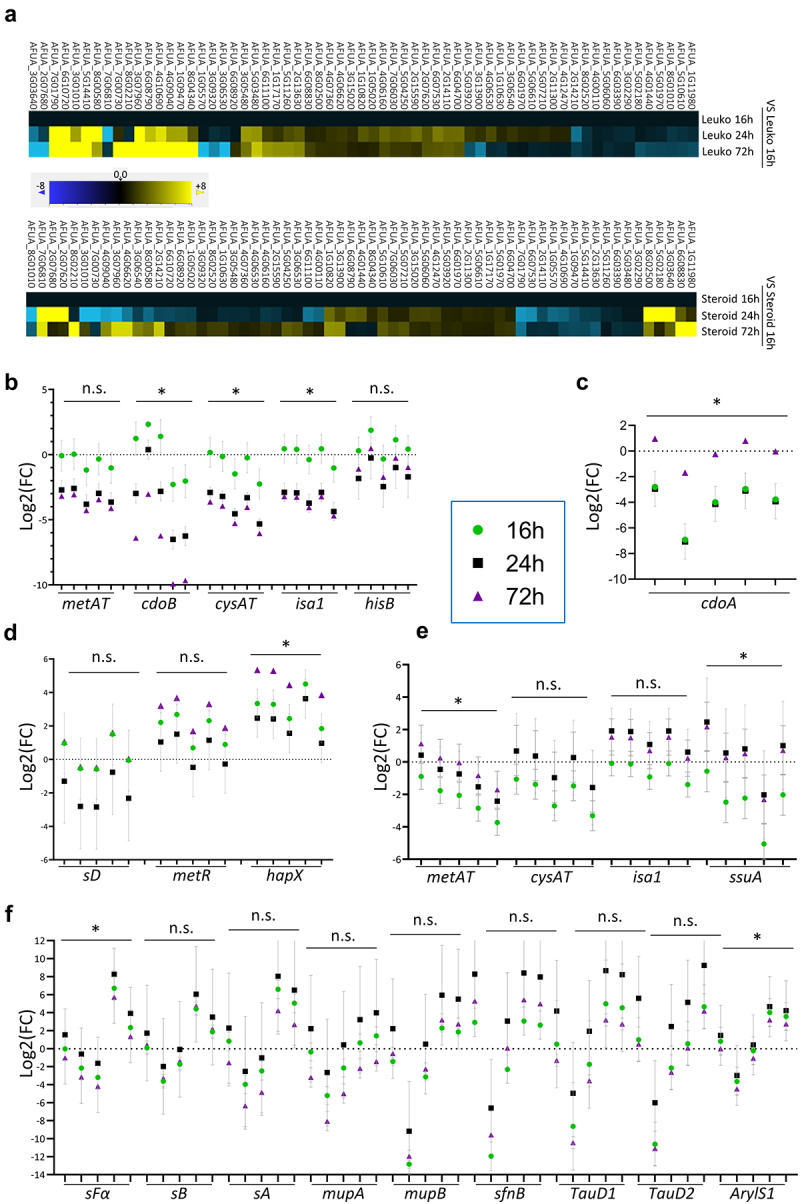
Temporal dynamics of gene expression reveals the existence of potential initiation and disease progression factors.

Since we found that the S-related metabolic status correlates with the time period post infection, we reasoned that some genes may be expressed at different levels as fungal infection progresses. To investigate this hypothesis, we first examined the fold change of the (geometric) mean expression of all genes at each time point with respect to the corresponding 16 h condition (Figure 3a). This analysis revealed that many genes changed the level of expression during the course of infection: transcription of several genes seemed to consistently increase, a few decreased in expression as infection progressed, and some appeared to increase or decrease in expression at mid-infection (24 h) before returning to initial levels in late infection (72 h). To explore whether any of these genes that showed temporal dynamic expression could be particularly important for *in vivo* growth, we analyzed the fold change of all genes in each *in vitro* condition versus each leukopenic (Fig. S2a) or steroid-treated (Fig. S2b) samples grouped by time after infection. As expected, we detected some genes that showed temporal variance *in vivo* compared with *in vitro*, as can be observed in Figures S2 and 3b-f. Although in many instances the differences in fold change *in vivo* versus *in vitro* among the 3 time-points were not statistically significant (one-way ANOVA), the patterns of temporal variance were maintained for all comparisons with *in vitro* conditions, suggesting that these detected genes do have a dynamic expression *in vivo* that may be particularly relevant for growth in the tissues. In detail, in leukopenic mice we observed that genes *metAT* (AFUA_2G13630), *cdoB* (AFUA_5G14410), *cysAT* (AFUA_1G09470), *isa1* (AFUA_4G10690), and *hisB* (AFUA_6G04700) had higher transcript levels *in vivo* at early (16 h) than at mid (24 h) or late (72 h) time points after infection (Figure 3b and Fig. S2a orange boxes), suggesting that these genes may be particularly important for the initiation of infection, but may not be as relevant for the progression and maintenance of infection. In contrast, *cdoA* (AFUA_1G05570) exhibited higher *in vivo* transcription during advanced infection (Figure 3cand Fig. S2a, purple box), suggesting that this gene may be relevant for growth in progressively invaded tissues. The genes *sD* (AFUA_1G10820), *metR* (AFUA_4G06530) and *hapX* (AFUA_5G03920) displayed higher expression at early (16 h) and late (72 h) time points after infection (Figure 3d and Fig. S2a black box), suggesting that they may be particularly important in initiating infection and during established infection, but that they are not crucial for infection progression.

a) Fold change of expression of genes in each time-point with respect to the correspondent 16 h condition revealed that most genes changed expression during the course of infection. The geometric mean value for each time-point was used. b–f panels display the Log2 fold change (FC) of genes that showed temporal dynamic expression in five randomly selected representative *in vitro* condition (in order: -N, -S, 0.1 mm Cys, 2 mm SH_2_, and 5 mm Met) versus *in vivo* conditions. All graphs display the mean and the standard error of the mean (SEM). Data were analyzed using one-way ANOVA with Tukey’s multiple comparisons test. Not significant = n.s.; * = *p*<0.05. b) These five genes always showed higher expression at early (16 h) than at mid- or late (24 or 72 h) infection of leukopenic mice when compared with the *in vitro* conditions. c) In leukopenic mice, the gene *cdoA* consistently showed the highest level of expression at late infection, when compared with the *in vitro* conditions. d) These three genes always showed higher expression at early and late (16 and 72 h) than at mid- (24 h) infection of leukopenic mice, when compared with the in vitro conditions. e) The four genes consistently showed higher expression at mid- and late infection than at early infection, when compared with the *in vitro* conditions. f) These nine genes always showed the highest level of expression at mid- (24 h) than at early or late infection, when compared with the *in vitro* conditions.

Similarly, in steroid treated mice, we observed that genes *metAT* (AFUA_2G13630), *cysAT* (AFUA_1G09470), *isa1* (AFUA_4G10690), and *ssuA* (AFUA_7G01790) had higher expression at mid- (24 h) and late (72 h) times after infection (Figure 3e and Fig. S2b purple box), indicating that they may be more relevant for the progression and maintenance of infection than for its initiation. Finally, genes *sFα* (AFUA_6G08920), *sB* (AFUA_1G05020), *sA* (AFUA_3G06540), *mupA* (AFUA_4G09040), *mupB* (AFUA_7G00730), *sfnB* (AFUA_8G01010), two taurine dioxygenase encoding genes (AFUA_3G0796 and AFUA_3G01010), and one arylsulfatase encoding gene (AFUA_8G02520) had higher expression *in vivo* at mid-infection time (24 h) (Figure 3g and Fig. S2b orange boxes), suggesting that these genes may play a role in this model for the progression of infection.

Therefore, we propose that the temporal dynamics of *in vivo* transcript levels may be useful for identifying and differentiating fungal factors of disease initiation and progression, defined as products that are essential for the initiation of infection or facilitate persistence and continued disease progression [36]. Although this hypothesis needs to be validated further by confirming the role of some of our identified candidates as true disease initiation or progression factors in appropriate models of infection, we emphasize the potential of *in vivo* transcriptomics at distinct stages of infection to detect such initiation and progression factors, as has already been proposed [36].

### Validation of an identified gene, encoding a serine hydroxymethyltransferase (SHMT), as a promising target for antifungal drug development

To demonstrate the value of NanoString technology in identifying genes relevant for *A. fumigatus* virulence, and aiming to validate a novel drug target, we decided to further investigate the serine hydroxymethyltransferase encoding gene (*shmB*, AFUA_3G09320), which was consistently expressed at higher levels *in vivo* compared with *in vitro* (Figure 1b). The *A. fumigatus* genome encodes two paralogs of serine hydroxymethyltransferase, ShmA (annotated as AFUA_2G07810, not included in the NanoString analysis) and ShmB. The ShmB protein contains 471 amino acids (aa) and ShmA is 537 aa in length, and both share an identity of 61.9% and similarity of 79.9% (Fig. S3a). Interestingly, ShmA has a long N-terminal region, which is absent in ShmB (Fig. S3a), which prompted us to investigate the subcellular localization of the proteins using three different public software programs: DeepMito [37], PProwler [38], and TargetP [39]. In contrast to the current annotation in the FungiDB and NCBI databases, all three algorithms predicted a mitochondrial localization for ShmA and a cytosolic localization for ShmB. Indeed, when we aligned *A. fumigatus* proteins with their *Saccharomyces cerevisiae* orthologs, we observed that the yeast mitochondrial enzyme SHM1 also had a longer N-terminus in comparison to the cytosolic proteins (Fig. S3b).

**Figure 4.**
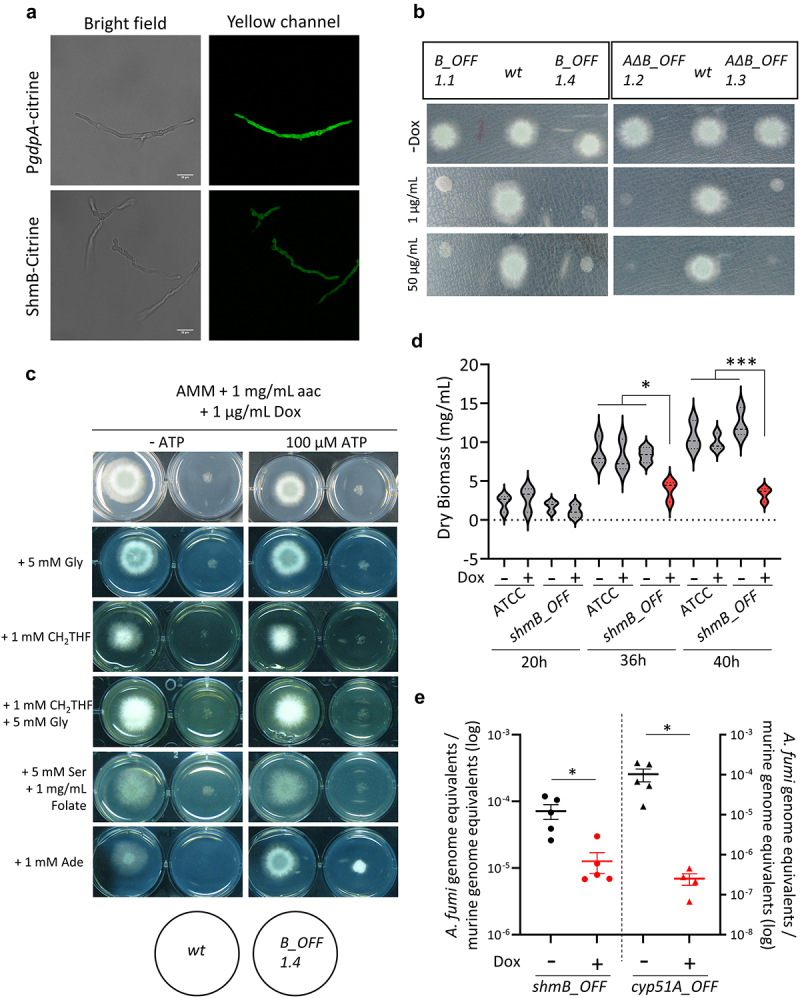
The cytosolic serine hydroxymethyltransferase is essential for viability and virulence in *Aspergillus fumigatus*.

Therefore, to clarify the subcellular localization of ShmA and ShmB in *A. fumigatus* we tagged the proteins at their C-terminus with the fluorescent YFP-derivative Citrine to express them under the control of their native promoters. In line with our predictions, we found that the ShmB-Citrine protein localizes throughout the cytoplasm, with a pattern similar to that of the control strain that expresses Citrine under the control of the strong *gdpA* promoter (Figure 4a). Regrettably, ShmA-Citrine could not be detected, possibly reflecting very low or no expression of the encoding gene under steady-state conditions, which is supported by its dispensability under standard laboratory conditions (see below).

a) A ShmB-Citrine tagged proteins localised diffused throughout the cytoplasm, similarly as the control P*gdpA*-Citrine strain. b) A Phenotypic assay with two independent clones of the *shmB_tetOFF* and *ΔshmA shmB_tetOFF* mutant strains. In the presence of doxycycline (Dox) the mutants did not grow, demonstrating that *shmB* is essential in *A. fumigatus*, and that ShmA cannot cover its function. Plates were incubated for 48 h at 37°C. The experiment was repeated twice independently. c) Attempting to reconstitute *shmB_tetOFF* growth in restrictive conditions (+Dox), media was supplemented with various metabolites which could potentially be limited in the absence of serine hydroxymethyltransferase activity. The only condition that could partly reconstitute growth, not at wild-type level, was supplementation with ATP and adenine (Ade). Plates were incubated for 48 h at 37°C. The experiment was repeated twice independently. d) Dox (10 µg/mL) was added or not to 12 h grown submerged mycelia of the *shmB_tetOFF* and its parental strains. Measurements of fungal biomass at 20 h, 36 h, and 40 h post-inoculation showed a significant reduction of growth after downregulation of *shmB*. The experiment was repeated three times independently, with three technical replicates. The results were analysed using a one-way ANOVA with Tukey’s multiple comparisons test. * = *p*<0.05 and ***=*p*<0.001. e) Fungal burden in the lungs of leukopenic mice treated or untreated with Dox. Downregulation of *cyp51A* (encoding the target of the azoles) expression caused a significant reduction in burden (*p* = 0.0159 in a nonparametric Mann Whitney test and *p* = 0.0476 in an unpaired *t*-test with Welch’s correction, run in GraphPad Prism 9). Similarly, downregulation of *shmB* triggered a significant decrease in fungal burden (*p* = 0.0159 in a nonparametric Mann Whitney test and *p* = 0.0288 in an unpaired *t*-test with Welch’s correction, run in GraphPad Prism 9). Therefore, ShmB appears to be a valid antifungal target. Each point represents an independent mouse (biological replicate/experimental unit) and the graph shows the mean and the standard of the mean (SEM). Comparisons were made using a Mann–Whitney nonparametric *t*-test.

To investigate the potential relevance of ShmA and ShmB in fungal virulence, we initially attempted to construct single null mutants with deletions in each encoding gene. We successfully constructed a *ΔshmA* mutant, which showed no phenotype under regular laboratory conditions, growing perfectly on rich Sabouraud and defined AMM media (Fig. S3c). In contrast, we were unable to generate a *ΔshmB* mutant, suggesting that this might be an essential gene in *A. fumigatus*. Therefore, we constructed two mutant strains in which we placed the *shmB* coding region under the control of the *tetOFF* module [15,40], one in the wild-type background and one in the *ΔshmA* background. The *tetOFF* system allows the downregulation of transcription of the gene of interest, in this case *shmB*, upon supplementation with doxycycline (Dox) [40]. The addition of Dox inhibited the growth of both strains (*shmB_tetOFF* and *ΔshmA-shmB_tetOFF*) on AMM solid plates (Figure 4b), proving that *shmB* is essential for cell viability independently of its paralog *shmA*. Gene essentiality is a conditional phenomenon, whereby particular growth/environmental conditions may overcome disturbances derived from gene deficiency [15,41–43]. Therefore, we attempted to reconstitute the growth of the *shmB_tetOFF* strain in the presence of Dox by supplementing compounds that could potentially be depleted in the absence of ShmB (Figure 4c). We previously observed that disturbances in the folate cycle causes a need of amino acid supplementation [44]; therefore, we assayed all phenotypic conditions on AMM without nitrogen (AMM-N, to diversify and increase the presence of permeases in the membrane that ensure the uptake of amino acids and other compounds) supplemented with 1 mg/mL of all proteinogenic amino acids. Additionally, we attempted to reconstitute growth by supplementing the media with 5,10-methylenetetrahydrofolate (CH_2_-THF) and glycine (separately and combined), or with folic acid and serine (substrates/products of the bidirectional enzymatic reaction), or with adenine (as blockade of the folate cycle might impair purine biosynthesis [45]). In addition, we tested the addition of ATP to all supplements, as we previously showed that impairing the folate cycle by blocking the activity of methionine synthase affects cellular energetics in *A. fumigatus* [15]. The only condition that could partly reconstitute growth, albeit not to the wild-type levels, was supplementation with adenine and ATP (Figure 4c). None of the supplement combinations reconstituted the growth of the *shmB_tetOFF* strain under restrictive conditions (Figure 4c). Therefore, either *shmB* is a truly essential gene or the conditions to overcome its essentiality are complex and yet unknown. Yet, based on these results *shmB* constitutes a promising drug target candidate, and consequently we decided to test its value in an *in vivo* infection model.

We recently proposed that potential drug targets should be validated in models of established infection, which is particularly relevant for essential genes (to confirm that essentiality cannot be overcome during active growth in the host tissues) [15]. To this end, we initially confirmed that Dox addition very strongly reduced down *shmB* expression in the growing mycelia (Fig. S3d) and that this downregulation caused a significant decrease in the growth capacity submerged mycelia grown for 12 h, as measured by fungal biomass (Figure 4d). Therefore, we assayed our optimized protocol to investigate targets in established *A. fumigatus* invasive pulmonary infections using the *tetOFF* system [15]. Leukopenic mice were infected with 2 × 10^5^ conidia of the *shmB_tetOFF* strain, Dox treatment was started 16 h after infection and fungal burden in the lungs was measured 72 h after infection. As a control, we also infected mice with the previously validated strain *Δcyp51B-cyp51A-tetOFF* [15], which expresses *cyp51A*, the gene that encodes the target of azole antifungals, under the control of the same *tetOFF* system. Therefore, this control strain serves to corroborate that downregulating a validated target (azoles are the gold standard therapy for invasive pulmonary aspergillosis) causes a significant reduction in fungal burden, as indeed we detected (Figure 4e). Interestingly, we found that the downregulation of *shmB* in the established infection model triggered a significant reduction in the fungal burden (Figure 4e). Hence, ShmB appears to be a valid target for the treatment of aspergillosis infections.

### Structural-based analysis of ShmB druggability

Human serine hydroxymethyltransferase (SHMT) is considered a promising target for anti-cancer drug development [46,47]; accordingly various inhibitors have been developed (for instance, see references in [48]). Therefore, we decided to test the antifungal potential of an inhibitor of the human SHMT (Hit-1 [49]) on *A. fumigatus*. We performed broth microdilution assays incubating the fungus in RPMI-1640 medium in the presence of increasing concentrations of Hit-1 (from 2 µg/mL = 4.29 µM to 512 µg/mL = 1.1 mM), and did not detect any antifungal activity of this compound (Table 2). As this could be due to the inability of Hit-1 to penetrate fungal cells, we also tested Hit-1’s antifungal activity in the presence of colistin. This antibiotic has been shown to permeabilize *Candida albicans* cells [50], and we have confirmed that it also permeabilizes *A. fumigatus* swollen conidia to the membrane impermeable dyes fluorescein isothiocyanate (FITC) and Zombie-Aqua (Fig. S4). However, Hit-1 did also not inhibit *A. fumigatus* growth in the presence of colistin (Table 2).

**Table 2.** Broth microdilution assays of colistin and Hit-1 showed that neither of these compounds inhibit growth at the tested concentrations.

	Minimum inhibitory concentration (MIC)
Colistin	>64 µg/mL
Hit-1	>512 µg/mL
Hit-1 + 2 µg/mL Colistin	>512 µg/mL
Hit-1 + 4 µg/mL Colistin	>512 µg/mL

**Figure 5.**
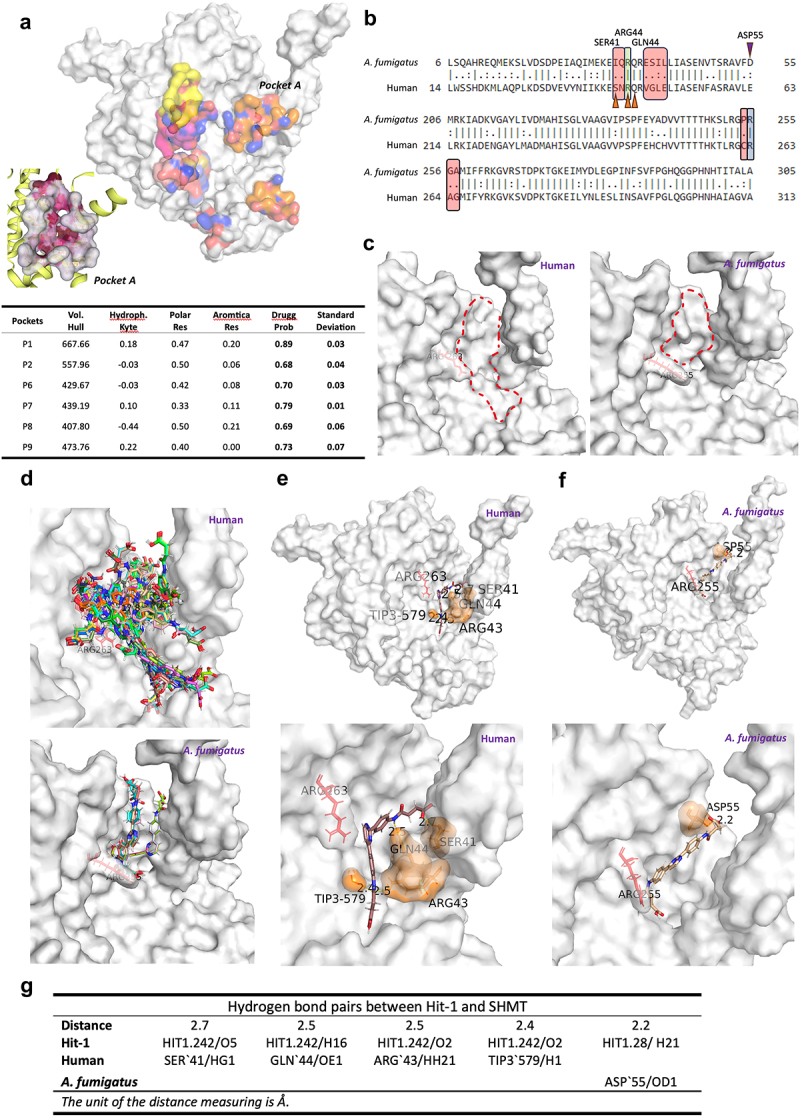
An inhibitor of the human SHMT does not bind properly to the fungal enzyme’s ligand binding pocket.

As our previous results with the *tetOFF* promoter demonstrated that ShmB is essential for *A. fumigatus* viability, we suspected that the inhibitor Hit-1, designed against the human enzyme, might not be effective against the fungal enzyme. This led us to hypothesize that it should also be possible to design fungal-specific inhibitors that are inactive against the human enzyme. To substantiate this hypothesis, our initial step involved identifying the main druggable binding pocket of human SHMT based on its crystal structure as retrieved from the Protein Data Bank (accession number PDB ID 1bj4), using a target-based *in silico* approach. We conducted two independent analyses employing the DrugRep [51] and the PockDrug [52] engines. The PockDrug analysis yielded 10 possible binding pockets, with only six of them possessing a Druggability Probability score exceeding 0.5 (Figure 5a). Cross-referencing with the results from DrugRep revealed a single common pocket from both predictions: pocket A (Figure 5a) (Gln44 Ala383 Ile51 Glu374 Phe56 Ser381 Ala379 Glu69 Val46 Arg43 Cys384 Ala407 Gly47 Glu49 Ile382 Arg465 Glu378 Glu54 Asn55 Leu48). Next, we investigated whether this predicted binding pocket is preserved in the *A. fumigatus* ortholog. To this purpose, we constructed a homological model of the fungal protein using the *in silico* protein modelling AI AlphaFold [53]. Although the similarity between the sequences is reasonably high (74.9%), indicating that the protein is well preserved across the species, there are some mutations in the amino acids within the key areas associated with the hypothetical binding pocket (highlighted in red in Figure 5b). A close comparison revealed that such mutations likely altered the conformation of the neighboring amino acid residues, resulting in a different topology of pocket A compared to that of human SHMT, making it much shallower and smaller (Figure 5c). In particular, the alteration in the position of Arg263 (Arg255 in *A. fumigatus* SHMT) formed a barrier resulting in the fungal pocket being almost half the size of the human pocket A.

a) Binding pockets with a druggability score above 0.5 predicted by DrupRep highlighted in human SHMT1 1bj4, with “Pocket A” Zoomed in (left corner). b) Alignment of amino acid sequence (partial) of *A. fumigatus* (ShmB) and human (SHMT1) cytosolic SHMT enzymes. Different amino acids (highlighted in red rectangles) were detected surrounding the key residues Arg263 in SHMT1 (Arg255 in ShmB, highlighted in blue rectangle); and Arg43 (Arg35 in ShmB, highlighted in green rectangle). Orange arrows indicate SHMT1 residues forming hydrogen bonds with Hit-1 and the purple arrow indicates the ShmB residue forming hydrogen bond with Hit-1. c) Comparison of predicted binding pockets between SHMT1 and ShmB with Arg263 (Arg255) highlighted in red. d) Hit-1 binding motifs suggested by SwissDock. e) Detailed interactions between Hit-1 and SHMT1. f) Detailed interactions formed between Hit-1 and ShmB. g) List of hydrogen bond pairs between Hit-1 and SHMT1 and ShmB.

Subsequently, we performed *in silico* docking using the SwissDock engine [54,55] employing the Hit-1 inhibitor (IC50 = 0.53 µM) [49] against pocket A of the human protein. This resulted in several promising binding motifs occupying the entire pocket, as visually represented in Figure 5d. For instance, in one of the top binding positions, HIT1.242 (ΔG = −7.57 kcal/mol; FullFitness = −2433.57 kcal/mol), Hit-1 nestled itself deep into the pocket, demonstrating hydrophobic interactions with the pocket (Figure 5e). Additionally, four hydrogen bonds were formed that were distributed throughout the molecular backbone (Ser`41, Gln`44, Arg`43 and Tip3`579; Figure 5G), anchoring the ligand neatly into the pocket (Figure 5e). Subsequent *in silico* docking between Hit-1 and the fungal ortholog pocket A suggested that Hit-1 could not bind as effectively. Only two binding motifs were suggested, both of which were restricted to the smaller *A. fumigatus* pocket (Figure 5d). Upon closer inspection of the top binding motif, HIT1.28 (ΔG = −6.87 kcal/mol; FullFitness = −2143.87 kcal/mol; Figure 5g), it is clear that the ligand is not embedding deep into the pocket, indicating less hydrophobic interactions with the target. There was only one hydrogen bond suggested between Hit-1 and the target (ASP`55; Figure 5f), indicating less favorable binding compared to the human version of the protein. It also appeared that the altered Arg255 acted as a barrier, blocking Hit-1 from extending to the region proximal to Arg43 (Arg35 in *A. fumigatus* SHMT) and forming additional hydrogen bonds (Figure 5f). Therefore, it appears that Hit-1 is specific to human SHMT, as it cannot bind properly to the fungal ortholog, which is in agreement with the observed absence of growth inhibition in *A. fumigatus* (Table 2). Similarly, we believe that inhibitors that bind to the fungal, but not the human, enzyme pocket can be designed. Thus, we propose that the development of novel fungal-specific inhibitor agents against the promising SmhB antifungal target is feasible.

## Discusion

New antifungal agents are urgently needed, as resistance to current drugs is widespread [56–58], posing a great threat to human health [25]. The selection of valid drug targets is crucial for the development of novel and efficient chemotherapy [59]. In the last few years, various innovative approaches have been applied to identify suitable target candidates. For instance, comparative genomics [60], computer modelling [61], *in silico* analysis of protein interactions [62], screening of deletion libraries [63], including competitive fitness assays [64], screening of regulatable libraries [43,65], construction of transposon insertion libraries [66,67], or machine learning approaches [68] have been employed to identify antifungal drug targets. In this regard, *in vivo* transcriptomics has also been used frequently to understand fungal pathogenicity [35,69–72], although its potential to identify suitable antifungal targets still encounters some skepticism. In a seminal study, McDonagh et al. [35] compared the transcriptome obtained from conidia germinating in murine lungs with germlings obtained under *in vitro* conditions expected to match the *in vivo* environment. This study confirmed that *A. fumigatus* encounters nutrient (particularly iron and nitrogen) limitations as well as alkaline and oxidative stress during the initiation of infection, providing foundational cues about the conditions encountered by the fungal pathogen in the mammalian lung. However, in order to detect fungal RNA, this study needed to use pools of 24 mice per sample, which limited the number of conditions that could be tested (for instance, only one model of immunosuppression was assayed), could only analyzed the early initiation of infection (12–14 h) and had to recover the fungal material from bronchoalveolar lavages. These limitations were mainly due to the challenge of recovering sufficient fungal RNA material from infected tissue for subsequent analysis. In a more recent study, NanoString analysis was employed to identify *A. fumigatus* transcriptional factors that are highly expressed or strongly upregulated during growth in murine lung tissues, and experimentally proved that two of them are relevant in the context of infection [73]. Here, we reasoned that comparing the transcription levels of genes related to sulfur metabolism in the tissues versus a variety of *in vitro* S-sources could reveal gene products that are specifically upregulated *in vivo*, and may therefore be relevant for the infection process and constitute promising antifungal targets.

A major advantage of our approach compared with previous studies on sulfur metabolism is that we could explore the potential relevance of many genes implicated in this process simultaneously. In fact, our analysis identified 15 genes that were consistently expressed at higher levels in murine lungs than in all tested *in vitro* conditions. At least five of these genes have been previously proven to be important for *A. fumigatus* virulence, and additionally six genes have been suspected to be relevant (Table 1). We previously showed that AFUA_5G02180, which encodes cysteine synthase (CysB), is important for *A. fumigatus* virulence, as a cysteine auxotroph has reduced virulence in a murine model of infection [14]. We have also demonstrated that AFUA_8G04340 encodes cystathionine-γ-lyase (MecB), the major persulfidating enzyme in *A. fumigatus*, which is relevant for fungal virulence [34]. The genes AFUA_2G07680, encoding L-ornithine-*N* ^5^-monooxygenase (SidA), which catalyses the first committed step of siderophore biosynthesis, and AFUA_5G03920, encoding the master transcriptional regulator for adaptation to iron limitation (HapX), have been shown to be crucial for virulence [29,30]. Finally, the gene AFUA_8G04340, encoding the transcriptional activator of the cross-pathway control system of amino acid biosynthesis (CpcA), was also reported to contribute to *A. fumigatus* virulence [31]. Therefore, we propose that the remaining genes are likely to be important for *A. fumigatus* virulence and thus constitute good candidates for experimental validation. Notably, the genes that we have characterized and shown to be dispensable for virulence (AFUA_1G05020 -*sB*- and AFUA_2G15590 -*sF*- [14]) were not detected in this analysis, further supporting the hypothesis that the genes identified may indeed be relevant. Nevertheless, it also needs to be mentioned that several genes that have been described as relevant for virulence in other studies were not identified as universally important in our analysis (for instance, AFUA_6G04700 -HisB-, AFUA_4G06530 -MetR- or AFUA_2G14210 - Ilv3A/IlvC- [13,27,74]), implying that our approach is not able to detect all genes relevant to infection. Yet, our analysis identified some of these genes as potential disease initiation or progression factors, which raises the question of whether their relevance for infection might be time-dependent.

In fact, our temporal analysis revealed various potential disease initiation and progression factors. These are defined as products required for the initiation of infection or the persistence and progression of the disease [36]. Differentiating these actions is paramount for drug discovery, as it would not be adequate to develop a drug to treat established infections that targets an initiation factor, and similarly, it would not be appropriate using a drug that targets a progressing factor as prophylaxis. Therefore, discerning disease initiation and progression factors is important to better understand the infective capacity of *A. fumigatus* and to expand our reservoir of potential antifungal targets. In fact, progression factors, which are usually not identified in classic virulence studies, are regarded as valuable targets, as antimicrobials are often employed to treat established infection [36]. In this respect, we previously negated a role of *sFα* (AFUA_6G08920) and *sB* (AFUA_1G05020) in *A. fumigatus* virulence by infecting leukopenic mice with null mutants [14]; however, our current results suggest that they might be disease progression factors specifically relevant to the steroid model (Figure 3c).

Hence, our study not only demonstrates that NanoString is a powerful tool to identify *A. fumigatus* genes important for its pathogenic potential but also provides various candidates to be investigated in future studies.

It was unfortunate that the samples from two leukopenic mice did not run properly and had to be excluded from the analyses. We acknowledge that this limits the value of some of our results, particularly the comparisons among time-points and the analysis of temporal expression of genes in leukopenic mice. However, as all infections for the *in vivo* samples of the analysis were carried out simultaneously, we reasoned that repeating just two mice would not provide fully consistent results. Indeed, we tried to include the data obtained from the two leukopenic mice of the preliminary experiment to validate the linear amplification protocol (Figure 1a), but found that these samples clustered slightly apart from the other *in vivo* samples (Fig. S5), and therefore could not be used for the model and temporal analyses. Importantly, including these two mice, the overall profile of expression relative to *in vitro* remained highly similar, such that the 15 genes previously detected to be consistently upregulated *in vivo* VS *in vitro* were still detected (Fig. S5), which demonstrates the strength of the results. With these two additional mice one more gene was included in the list of candidates, AFUA_5G07210. This gene encodes a homoserine O-acetyltransferase, which is important for virulence in the fungal pathogen *Cryptococcus neoformans* [75].

One of the uncharacterized genes in our list of 15 potentially relevant genes encodes serine hydroxymethyltransferase (SHMT). SHMT is a pyridoxal phosphate-dependent enzyme in the folate cycle that catalyzes the reversible conversion of serine and tetrahydrofolate (THF) into glycine and 5,10-methylenetetrahydrofolate (CH_2_-THF). Fungi encode two isoforms in the nuclear genome: one cytosolic and one mitochondrial [45]. In *S. cerevisiae* both genes were disrupted singly and in combination, generating auxotrophic viable strains [76]. In contrast to our results, the deletant of the cytosolic SHMT encoding gene (*SHM2*) grew well in the presence of the substrates glycine and formate. In addition, the cytosolic and mitochondrial SHMT encoding genes could also be deleted in the ascomycete *Ashbya gossypii* [77], where supplementation with adenine or glycine could reconstitute the growth of the cytosolic *SHMT* (*SHM2*) knock out. Therefore, the essentiality of cytosolic ShmB in *A. fumigatus* suggests that the metabolic implications of disrupting one-carbon cycle are more complex for this pathogen than for other fungi. Indeed, we previously showed that in contrast to other fungi, the absence of methionine synthase, which forms a junction between the one-carbon cycle and the trans-sulfuration pathway, causes an imbalance in cell energetics, possibly initiated by cellular sensing of purine depletion, which halts fungal growth [15]. Here, we found that adenine could partly reconstitute the growth of the *shmB_tetOFF* strain in restrictive conditions, which resembles the phenotype we observed with *metH_tetOFF* [15]; however, in contrast to the absence of MetH, ATP could not fully reconstitute the growth in the absence of ShmB, indicating that we do not understand the metabolic implications of eliminating cytosolic serine hydroxymethyltransferase activity. Given its essentiality, we hypothesized that it could be a good target for the development of novel antifungals, and indeed we have validated it in a murine model of established infection using the *tetOFF* system. To our knowledge, the relevance of serine hydroxymethyltransferases in fungal pathogenicity has not been previously investigated. In contrast, this enzyme has been shown to be a promising antimalarial target [78] and is implicated in a variety of virulence-related traits in bacteria. For instance, a null mutant of *Staphylococcus aureus* showed impaired survival inside macrophages and reduced virulence in a *Galleria mellonella* infection model [79]. In *Helicobacter pylori*, deletion of the SHMT encoding gene *glyA* caused reduced growth rate [80], and in *Pseudomonas aeruginosa* controls rugose colony morphology, increases biofilm formation, abolishes swarming and is related to the redox status of the cells and the control of iron acquisition [81]. Therefore, the targeting of microbial SMHT enzymes is a promising antimicrobial strategy. Nevertheless, as current SHMT inhibitors have been developed against human enzymes, specific inhibitors of microbial proteins need to be designed. By comparing the pocket landscape of *A. fumigatus* and human enzymes and performing a docking assay with a known inhibitor of the human SHMT, we showed that there are sufficient differences to pursue the development of a fungal-specific agent. A comparable situation occurs in the case of efforts to inhibit the promising antifungal target calcineurin. Human and fungal enzymes are highly similar, such that in this case a drug developed against the human protein (FK506) can inhibit the fungal enzyme and is a potent antifungal agent. Fueled by structural insights, recent research has focused on developing FK506 fungal-specific derivatives, which have already generated promising results [82–84].

In conclusion, in this study we showed that *in vivo* transcriptomics is a valid strategy for identifying virulence traits in *A. fumigatus*. Using NanoString technology we detected 15 genes that are potentially relevant for pathogenicity, five of which have already been proven experimentally. We further investigated a novel gene, encoding cytosolic serine hydroxymethyltransferase, thereby demonstrating that this is an essential gene product for *A. fumigatus* viability and virulence, and that it seems possible to design specific inhibitors for the fungal enzyme. Therefore, we validated cSHMT as a promising antifungal target for future studies.

## Material and methods

### Fungal strains and culture media

The *Aspergillus fumigatus* strain ATCC46645 [85] was used for transcriptomic analysis, and the MFIG001 isolate (*ΔKU80*) for genetic manipulation [86]. Both strains were obtained from the MFIG strain collection repository. The strains were routinely grown on Sabouraud solid medium (Oxoid) for 3–5 days at 37°C for spore harvesting.

For phenotyping analysis, the isolates were inoculated on solid *Aspergillus* Minimal Medium (AMM, 1% glucose, 5 mm ammonium tartrate, 7 mm KCl, 11 mm KH_2_PO_4_, 0.25 mm MgSO_4_, 1× Hutner’s trace elements solution [pH 5.5], 1.5% agar), supplemented as indicated in each section, and incubated at 37°C for 2–3 days. Phenotypic experiments were independently performed at least twice.

For selection in the presence of resistance markers 50 μg ml^−1^ of hygromycin B or 100 μg ml^−1^ of pyrithiamine (InvivoGen) was added to the AMM in the growth plates.

### Mutant construction

A. *fumigatus* was transformed using a standard protoplasting protocol, as described in detail in [87].

To generate the *ΔshmA* mutant and attempt to construct the *ΔshmB* mutant, we followed a previously optimized protocol [87]. Briefly, upstream and downstream fragments of the genes were amplified by HIFI-PCR (Phusion DNA polymerase, ThermoFisher Scientific) and fused to the hygromycin-B (*hph*) selective cassette by fusion PCR (Fig. S6a). This deletion cassette was used to replace the target gene by homologous recombination in the *ΔKU80* strain MFIG001.

To construct the *shmB_tetOFF* isolate, we followed CRISPR-Cas9 mediated recombination, as previously described for *A. fumigatus* [88]. Briefly, Alt-R® *Streptococcus pyogenes* Cas9 V3 protein, a 67 mer Alt-R® CRISPR-Cas9 tracrRNA and locus-specific Alt-R® CRISPR-Cas9 crRNA (GTCCAAATGAAAGAAAACAA|TGG, designed using EuPaGDT [89]) (Integrated DNA Technologies) were assembled *in vitro* (as described in [88]) and delivered into *A. fumigatus MFIG001* protoplasts to create double-strand breaks right before the ATG start codon (Fig. S6b). A repair template, containing the *tetOFF* module and a pyrithiamine resistance marker (PtrA), was amplified from pSK606 [40] using the Phusion Green Hot Start II high-fidelity PCR master mix (ThermoFisher Scientific). This repair template had 50 bp flanking regions homologous to the upstream and downstream sequences at the ATG start codon, placing the *tetOFF* module immediately 5”- of the *shmB* ORF (Fig. S6b).

To construct the fluorescent SHMT strains, we followed the same CRISPR-Cas9 mediated recombination strategy [88], using the locus-specific Alt-R® CRISPR-Cas9 crRNAs GACAGGCAGGGGGTAGGTGC for *shmB* and GACAGGCAGGGGGTAGGTGC for *shmA* to create double-strand breaks at the 3’ gene-ends, in frame with the respective ORFs (Fig. S6c). The Citrine sequence was amplified from pGWKS5 [90] and replaced the *hph* coding region of the *hph* cassette on pAN7.1 [87] via Gibson assembly to construct the pCitrine*-hph* plasmid. The *PgdpA*-Citrine-*TtrpC* cassette was amplified by PCR and inserted into the pSK606 [40] plasmid to fuse it to the PtrA cassette, constructing the pCitrine*-ptrA* plasmid. The module Citrine-*TtrpC-PtrA* was amplified from this plasmid as repair template. Each repair template had 50 bp flanking regions homologous to the upstream and downstream sequences at the stop codon of the targeted gene (*shmA* or *shmB*) so that the Citrine tagged proteins were transcribed by their native promoters (Fig. S6c). To express the Citrine cytosolically, the *PgpdA*-Citrine-*TtrpC-PtrA* region was amplified from the plasmid by PCR and inserted into the Aft4 “safe haven” region [91].

All primers used in the study can be found in Table S3.

### Localization studies of ShmB-Citrine

*A. fumigatus* conidia were inoculated in 200 μL of filtered autoclaved AMM on 8 well chambers slides (IBIDI) and incubated at 30°C for 16 h. Images were acquired using a Leica SP8× laser scanning confocal microscope equipped with a 63× (NA 1.4) HC PLAN APO CS2 oil immersion objective. The pinhole was set to one Airy unit and the gain was set to 200. To visualize the localization of labelled proteins, fluorescence imaging was performed using excitation at 516 nm and emission a 529 nm using a white light laser. The image format capture was 1024 × 1024at scan speed of 400 hz. Z-stacks of germlings were taken at 1.0 microns and slices were projected as average intensity using ImageJ, and no adjustments to brightness or contrast were made. Scale bars are shown.

### Murine infections

Mouse infections experiments were performed under the United Kingdom Home Office project license PDF8402B7 and approved by the University of Manchester Ethics Committee and the Biological Services Facility at the Faculty of Biology, Medicine and Health, University of Manchester We have adhered to the ARRIVE guidelines for experiment design, execution, and report.

Outbred CD1 male mice (22–26 g) were purchased from Charles Rivers and allowed to rest for at least one week before the experiment. The mice were allowed access to water and food *ad libitum* throughout the experiment and had environmental enrichment (cardboard tube, shredded paper, and wooden blocks). Mice were allocated randomly to cages (one cage per group) before the groups were generated. Group allocation was performed by a blind investigator. For all treatments, the order of administration was random.

For the leukopenic model groups of three mice were immunosuppressed with 150 mg/kg of cyclophosphamide (Baxter) on days −3 and −1 plus 1 subcutaneous injection of 250 mg/kg hydrocortisone 21-Acetate (Sigma-Aldrich) on day −1. For the corticosteroid model, groups of three mice were injected with a single dose of 40 mg/kg triamcinolone (Bristol Myers Squibb) on day −1. Bacterial infections were prevented by adding 2 g/L neomycin to drinking water. Mice were anesthetized by exposure to 2% to 3% isoflurane (Sigma-Aldrich) for 5–10 min and intranasally infected with 40 μL of a suspension containing 10^5^ spores for the leukopenic model and 10^6^ spores for the corticosteroid model. For both models, three mice per time point post infection (16, 24, or 72 h) were used. At the designated time-points, mice were sacrificed by a lethal injection of pentobarbital and the lungs were harvested and immediately snap frozen for RNA isolation. A control group consisting of two leukopenic not infected mice was included (to confirm no cross-reactivity of murine and fungal RNAs in NanoString).

For the chronic model, we followed a published protocol [26,92]. Briefly, one immunocompetent mouse was anesthetized by intraperitoneal injection of ketamine (1%)/xylazine (0,2%) solution and intratracheally inoculated with 50 µL of a suspension of agar beads containing 5 × 10^7^ conidia/mL. The infection was allowed to progress for 21 days before the mouse was sacrificed for further processing.

The total number of mice used in the NanoString experiments was 23 (2 linear amplification + 9 leukopenic+ 9 corticosteroid + 1 chronic + 2 control).

To monitor the virulence of the *shmB_tetOFF* strain, we followed our previously optimized protocol [15]. Briefly, groups of 10 mice were infected with 40 μL of a suspension containing 2 × 10^5^ conidia of the *shmB_tetOFF* or the control *Δcyp51B-cyp51A-tetOFF* strain (inocula were confirmed by plating dilutions of the used suspensions). Five mice per strain were treated with doxycycline (Dox) and five with PBS vehicle. Dox treatment started 16 h after infection by an intraperitoneal injection (50 mg/kg) and change to Dox-containing food (Envingo Safe-Diet U8200 version 0115 A03 0.625 g/kg Doxycycline Hyclate pellets). The treatment was maintained by subcutaneous 50 mg/kg Dox injections every 12 h post infection until the end of the experiment after 72 h. Treatments were administered by a blinded investigator (not knowing the expected outcome of the experiment). Similarly, analysis of the fungal burden was carried out blind (identity of each group not revealed to the investigator). The total number of mice used in this experiment was 20.

The total number of mice used in this study was 43.

### NanoString analysis: preparation of samples, hybridization, and data processing

RNA was isolated from *in vitro* conditions as previously described [13]. Briefly, mycelia were transferred from a culture grown overnight in AMM to new flasks containing the defined sources described in Table S2. Mycelia were incubated for 4 h in the new conditions, then filtered through sterile Miracloth (Merck Millipore) and snap frozen in liquid nitrogen. Mycelia were ground in the presence of liquid nitrogen, and RNA was isolated using the RNeasy Plant Mini Kit (Qiagen) following the manufacturer’s instructions and including the on column DNAse digestion step. RNA quality and concentration were was assessed using a NanoDrop 2000 spectrophotometer (Thermo Fisher Scientific).

RNA from murine lungs was isolated as follows: lungs were lyophilized for 48 h in a CoolSafe ScanVac freeze drier connected to a VacuuBrand pump and subsequently ground in the presence of liquid nitrogen. The resulting powder was resuspended in TRIzol reagent (Invitrogen – Thermo Fisher Scientific) and processed according to the manufacturer’s instructions. RNA was purified with Phenol:Chloroform:Isoamyl (25:24:1) (Sigma) and extracted with chloroform (Invitrogen). Subsequently, RNA was purified using the RNeasy Minikit (Qiagen), following the clean-up protocol, and included the on-column DNAse digestion step.

RNA isolated from murine lungs was subjected to the NanoString nCounter Single Cell Gene Expression protocol for linear amplification of target genes. Briefly, RNA was converted to cDNA using the SuperScript VILO Master Mix (Life Technologies). To confirm that the target RNA was present in the samples and that cDNA was properly generated, Real-Time PCR was performed using the gene AFUA_4G07360 (*metH*) as a target, as RT-PCR for this transcript had been validated before [15]. Genes of interest were linearly amplified using Multiplex Target Enrichment (MTE) primers (Supplementary File 1) and a PCR protocol consisting of 16 MTE amplification cycles of 94°C 15 s and 60°C 4 min, with the TaqMan PreAmp Master Mix (Life Technologies).

cDNA samples were incubated for 2 min at 94°C and then snap-cooled on ice prior to addition to the hybridization reaction. Hybridization was performed according to the Elements Tagset Hybridization setup, as detailed by NanoString and using the NanoString custom designed probes A and B (Supplementary File 1). The cartridge was read using the flex system, consisting of the nCounter Prep Station and the Digital Analyzer.

The raw data file (Supplementary File S2) was initially subjected to Quality Control. This consisted of monitoring the counts of the internal positive control (positive performance for hybridization verification) and the total counts detected for each condition (Fig. S1).

Raw data (Supplementary File 2) were normalized using nSolver software (version 4.0; NanoString). Data were first subjected to Background Subtraction using the geometric mean of the negative control counts. Subsequently, the data were normalized using both the geometric mean of the positive control count and geometric mean of the housekeeping genes (Supplementary File 3).

Normalized data were analyzed using the agglomerative vluster – heat map option of the nSolver software (version 4.0; NanoString). Spearman’s Correlation was used as the distance metric and the average was used as the linkage method.

### Computational structure analyses

The crystal structure of serine hydroxymethyltransferase (SHMT) was obtained from the Research Collaboratory for Structural Bioinformatics Protein Data Bank (RCSB PDB, http://www.rcsb.org) in PDB format, referenced by the Protein Data Bank ID 1bj4. The structure of fungal SHMT was predicted using AlphaFold2 [53]. The amino acid sequence was retrieved from the FungiDatabase [93] and utilized as input, with the highest scoring model (pLDDT = 96.9, pTMscore = 0.943) selected for downstream *in silico* analysis.

For human SHMT, DrugRep [51] and PockDrug [52] were employed for target-based binding pocket prediction. DrugRep is a receptor-based screening tool that utilizes CB-Dock to identify docking pockets of the protein receptor. PockDrug utilizes the Fpocket estimation method, based on holo or apo proteins, for predicted pocket estimation. This method involves the preliminary detection of cavities capable of binding a ligand of sufficient size, without ligand proximity information. Pockets with a DrugRep predicted druggability score above 0.5 were recorded and cross-referenced with suggestions from PockDoc to determine the “optimal” binding pocket for subsequent *in-silico* docking.

Ligand Hit-1 was constructed using Marvin JS (https://marvinjs-demo.chemaxon.com/latest/demo.html [94]). The binding mode of Hit-1 to the optimal binding pocket in SHMT was predicted using the EADock DS-based SwissDock (http://www.swissdock.ch/docking# [55]). The binding modes with the most favorable energies were evaluated with Fast Analytical Continuum Treatment of Solvation (FACTS) [95] and then output as clusters. The best docking pose, exhibiting maximal hydrophobic pocket coverage and hydrogen bonding, was manually selected. Outputs from all programes were visualized in PyMOL 2.5 (Schrödinger, LLC).

## Supplementary Material

Table S3.docx

Table S1.xlsx

Supplementary Figure 1.tif

Supplementary File 2_RAW Data.csv

Supplementary Figure 3.tif

Supplementary Figure 4.tif

Table S2.xlsx

Supplementary File 1_Probes and MTE primers.xlsx

Supplementary Figure 2.tif

Supplementary File 3_Normalized Data.xlsx

Supplementary Figure 5.tif

Supplementary Figure 6.tif

Author Checklist Completed.pdf

## Data Availability

All NanoString data can be found at the Zenodo repository https://zenodo.org/records/13120359.
