# Supplementary material for: The sulfur-related metabolic status of Aspergillus fumigatus during infection reveals cytosolic serine hydroxymethyltransferase as a promising antifungal target
Source: Virulence. 2025 Jan 17;16(1):2449075. doi: 10.1080/21505594.2024.2449075 (PMC11749473; doi:10.1080/21505594.2024.2449075)
Supplement: Table S3.docx [file KVIR_A_2449075_SM1450.docx]

**Table S3**. Primers used in this study

|  | Description | Sequence 5’ 🡪 3’ |
| --- | --- | --- |
| *Hph* cassette | Hph_F | CCGGCTCGGTAACAGAACTANNNNNNNNNNGCCNNNNNNNNNNCAGAACGGCGTAACCAAAAGTCAC |
|  | Hph_R | GTTGGAGCATATCGTTCAGAGCNNNNNNNNNNTAGNNNNNNNNNNTTCATCTTGACGACCGTTGATCTG |
| shmA deletion AFUA_2G07810 | P1 | GTTCGCATGCCTGTTTGAGG |
|  | P2 | TAGTTCTGTTACCGAGCCGGTGTCAGGAAAAACGGGGTGA |
|  | P3 | GCTCTGAACGATATGCTCCAACGCTTCTTTTGGCGGGTTAGC |
|  | P4 | AGCTCGTCACTTGTGAGCTG |
|  | P5 | TCACGTTGCAGAGTCCTGAC |
|  | P6 | GCGTTATACGGATCGCCCA |
| smhB deletion (attempt) AFUA_3G09320 | P1 | TGGCCCTAAGAGTATCGCCT |
|  | P2 | TAGTTCTGTTACCGAGCCGGGAGAAGGGGAGAGAGGAGGA |
|  | P3 | GCTCTGAACGATATGCTCCAACCAGGCTTTTGACGAGTTCAACA |
|  | P4 | CCCCTACGTCAAGCTCCATG |
|  | P5 | CCCAGACTCTCGTTGGTGTC |
|  | P6 | GTTCTCACGACTCTTCCCCG |
| *shmB_tetOFF* Construction | shmB_tetOFF crRNA | GTCCAAATGAAAGAAAACAA |
|  | Tet-OFF Fw | GCCCAACCTTTTTTCCTCCTCTCTCCCCTTCTCTCTTCATCCCTATCATCTTCTAGAATGCCCCACCGTT |
|  | Tet-OFF Rv | GTGGGATGATTCACCTCACGGTGAGCCTGCGACAAAGCGTAGGTGGCCATGGTGATGTCTGCTCAAGCG |
| *shmB_tetOFF* validation | V1-OFF-Fw | TTCGACGGAAGACTATCTCG |
|  | V1-OFF-Rv | ACGTTCTCAGAGGCAATCAG |
| *shmB* RT-PCR | Fw | CGTCTCATGGGTCTGGATCT |
|  | Rv | TGCCGGTCTCAGTGTTAACA |
| Citrine insertion | shmB crRNA | GACAGGCAGGGGGTAGGTGC |
|  | shmB_C_Fw | CTCTCCGCAAGGAGGTCGCCGAGTGGGCTAGCACCTACCCCCTGCCTGTCATGGTCTCCAAGGGCGAGGA |
|  | shmB _C_Rv | AGAAAAGCATATTTTGGTATCAAGTGAGATTATTTGTTCTGCCCTGATGCGCCAATTGATTACGGGATCC |
|  | shmA crRNA | GGGCACTTTCAGCCTTCCGT |
|  | shmA_C_Fw | GAAAGGAGGTGGAAGACTGGGTGGGCACTTTCAGCCTTCCGTGAGATGATATGGTCTCCAAGGGCGAGGA |
|  | shmA_C_Rv | GAAGCCCTCAAAACATCATCTTTTCATTAAATAGCGGCAATGTATACTTTGCCAATTGATTACGGGATCC |
| Mcitrine insertion validation | V2_B_Fw | GTATGAGCGAGGAGGACTTC |
|  | V2_B_Rv | TGAAGATGAGAAGCTGGGAG |
|  | V2_A_Fw | TCCTTGAACTCTGTGGTGTC |
|  | V2_A_Rv | CAAATAAACAGCCTCACCCG |
