# Supplementary figures and images for: The sulfur-related metabolic status of Aspergillus fumigatus during infection reveals cytosolic serine hydroxymethyltransferase as a promising antifungal target
Source: Virulence. 2025 Jan 17;16(1):2449075. doi: 10.1080/21505594.2024.2449075 (PMC11749473; doi:10.1080/21505594.2024.2449075)

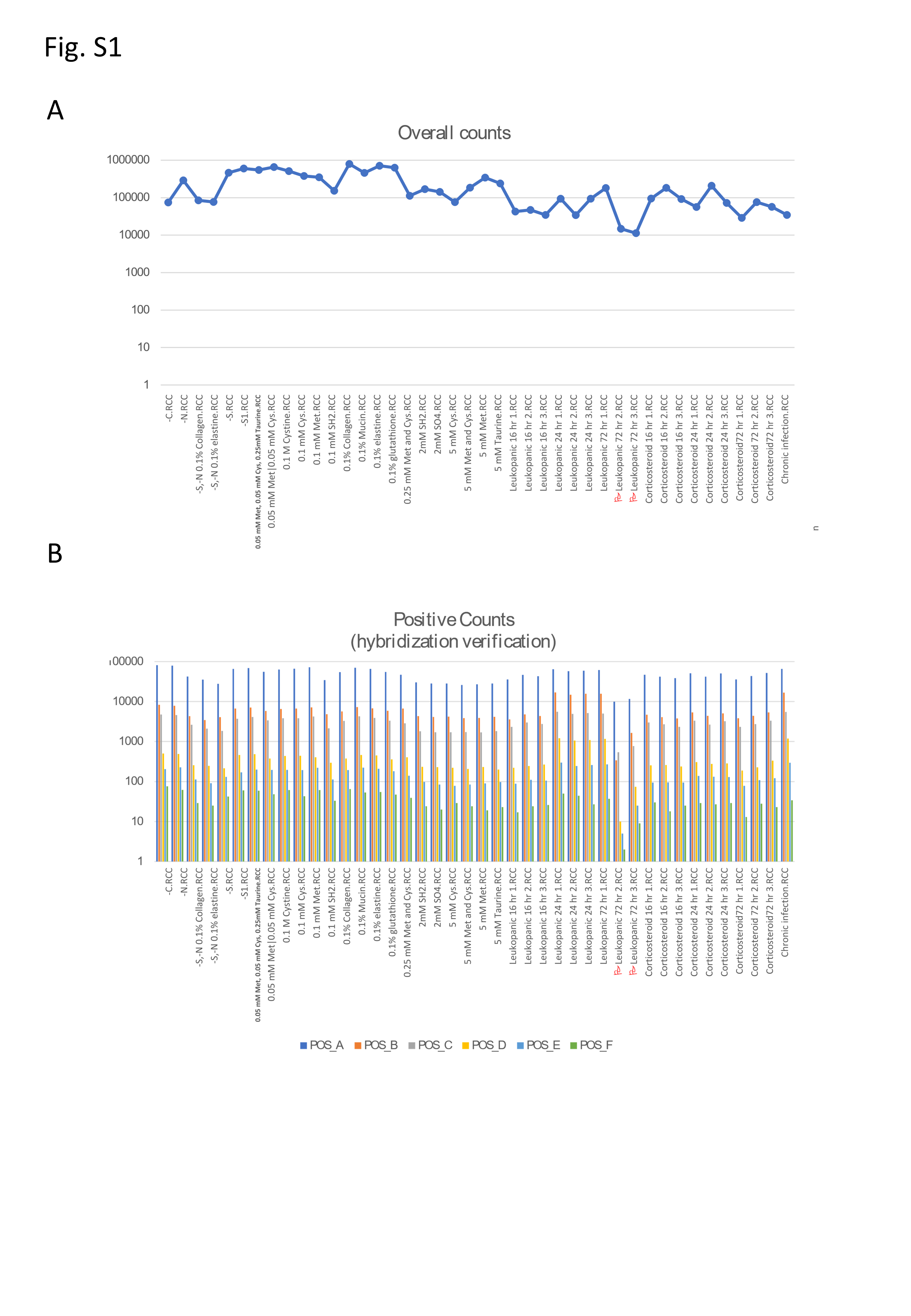

Supplement: Supplementary Figure 1.tif [file KVIR_A_2449075_SM1448.tif]

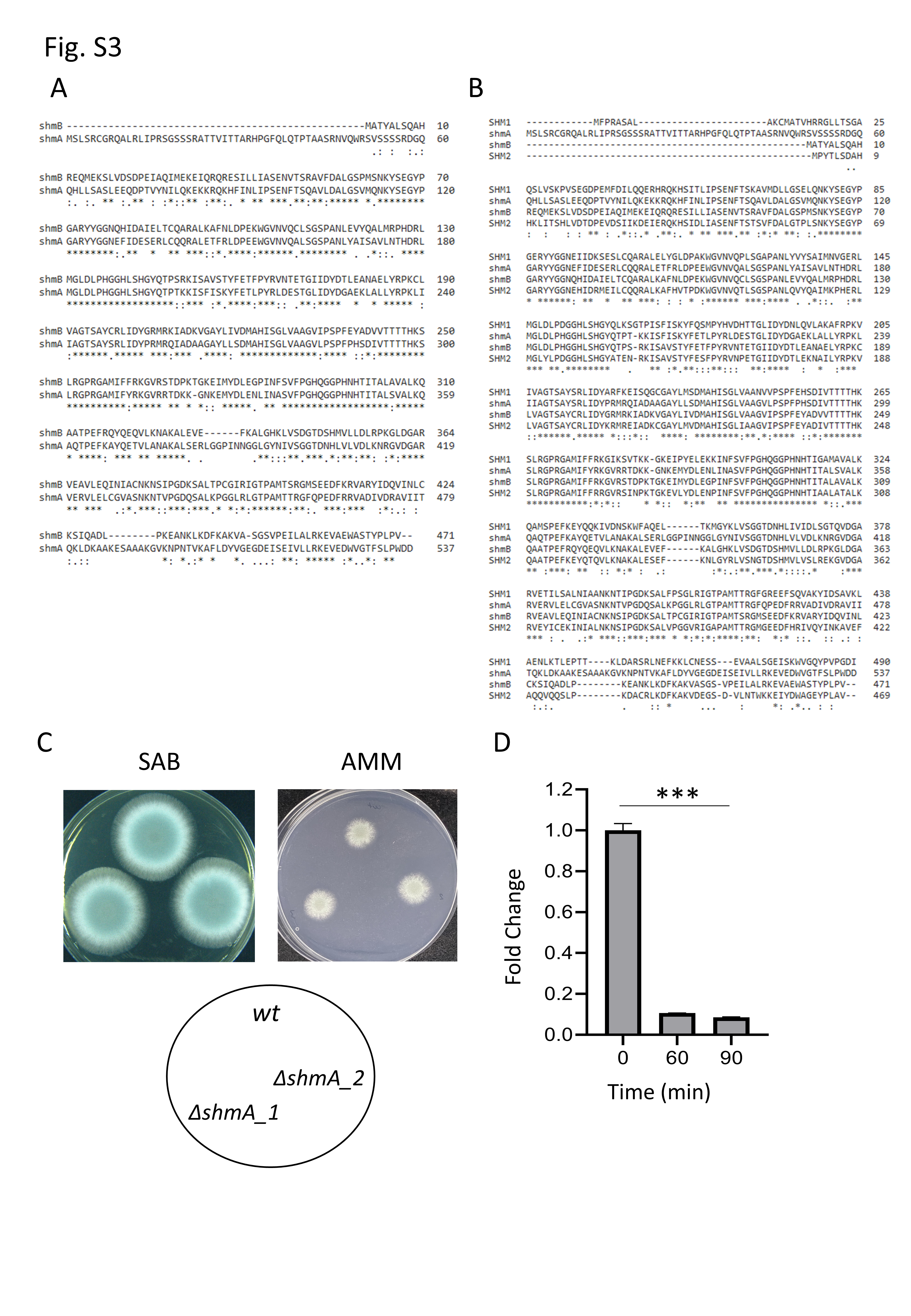

Supplement: Supplementary Figure 3.tif [file KVIR_A_2449075_SM1446.tif]

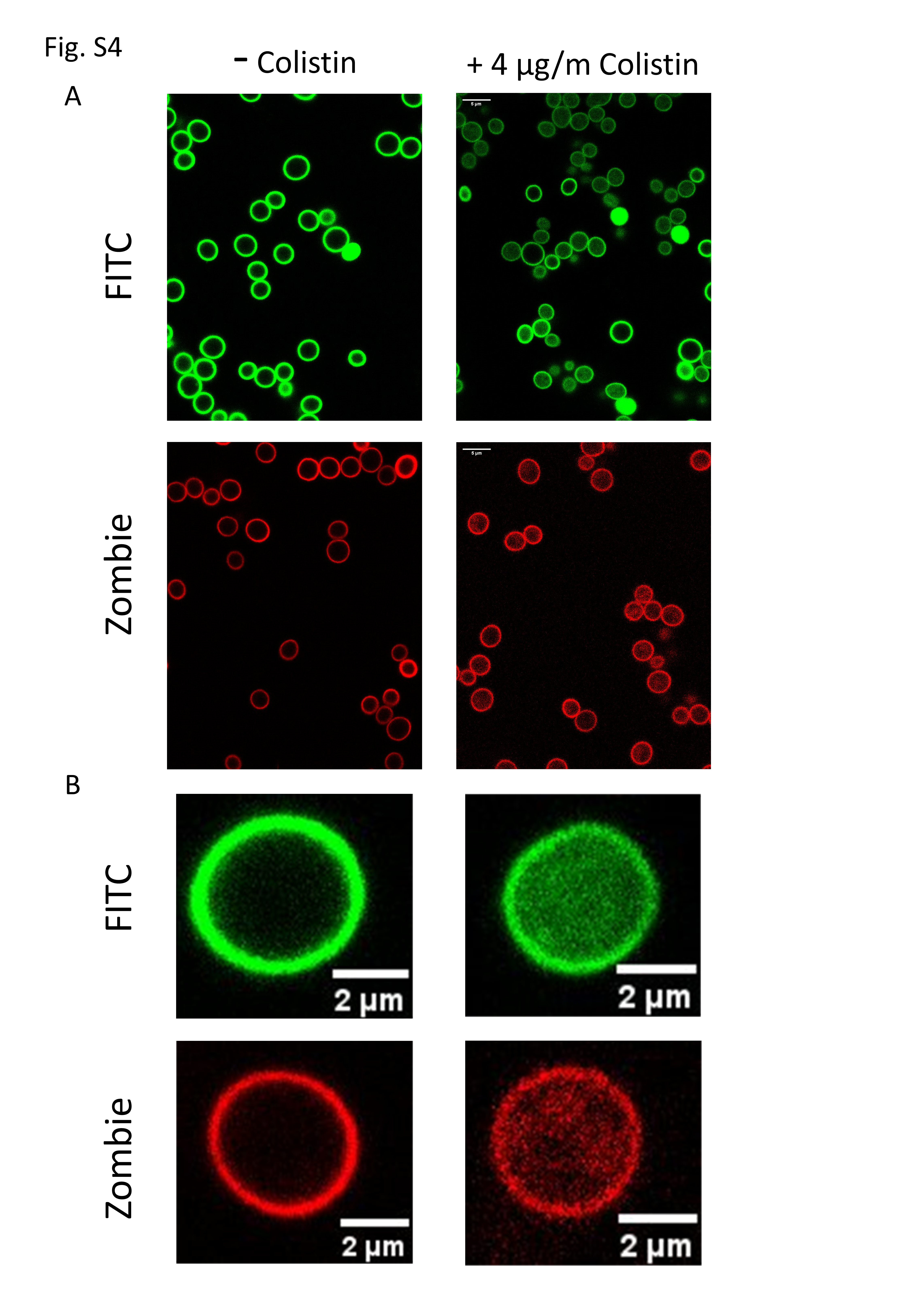

Supplement: Supplementary Figure 4.tif [file KVIR_A_2449075_SM1445.tif]

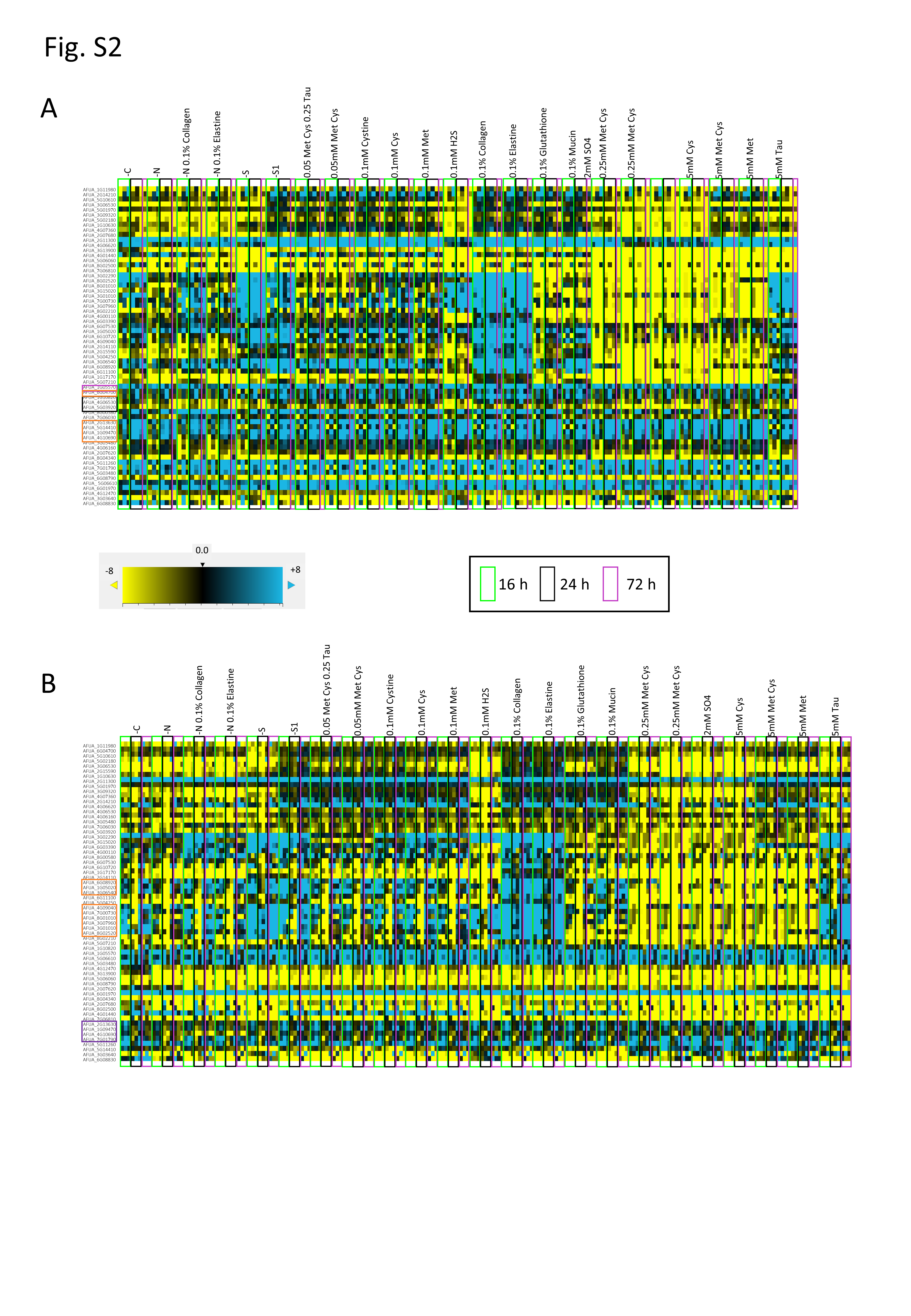

Supplement: Supplementary Figure 2.tif [file KVIR_A_2449075_SM1442.tif]

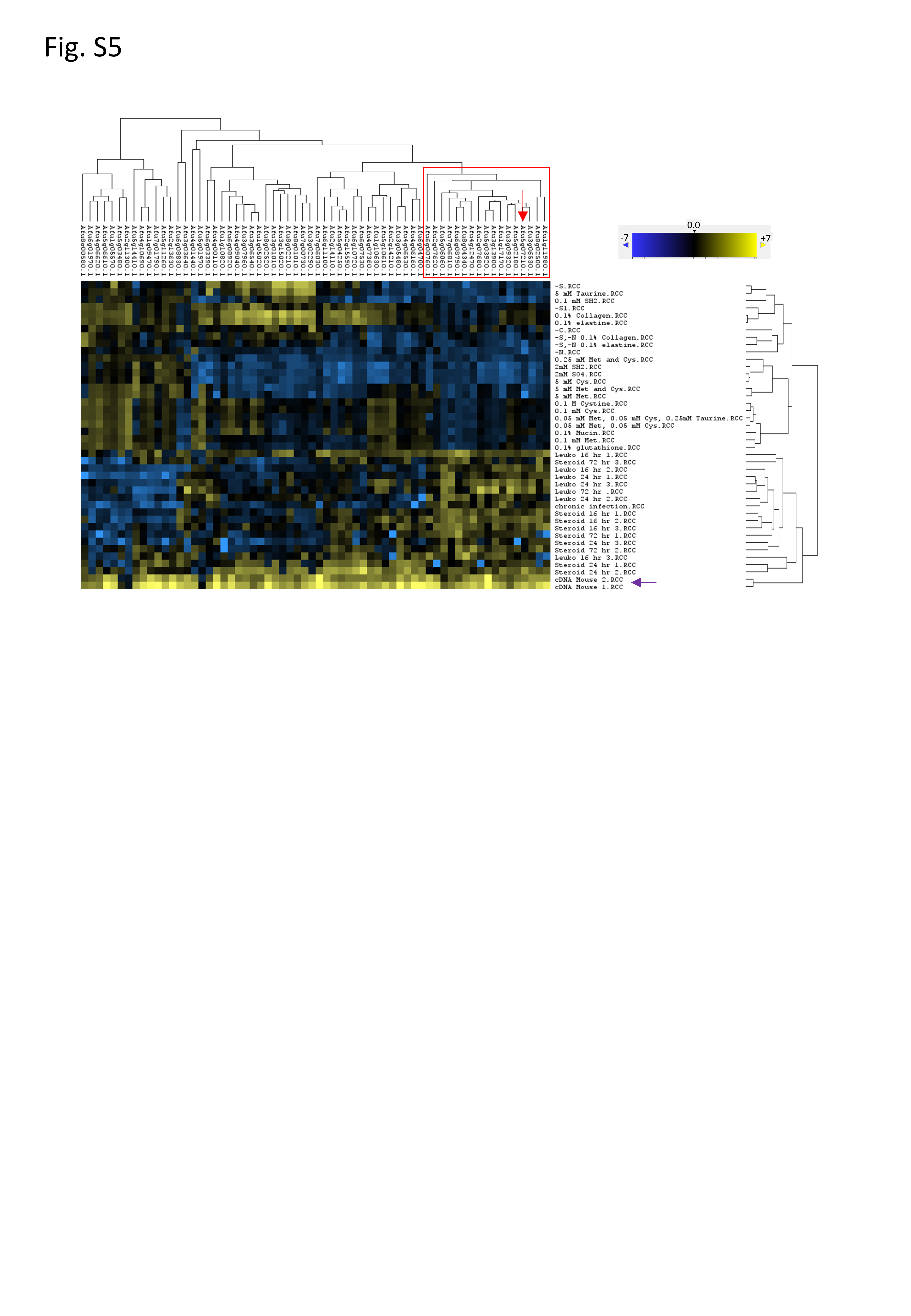

Supplement: Supplementary Figure 5.tif [file KVIR_A_2449075_SM1440.tif]

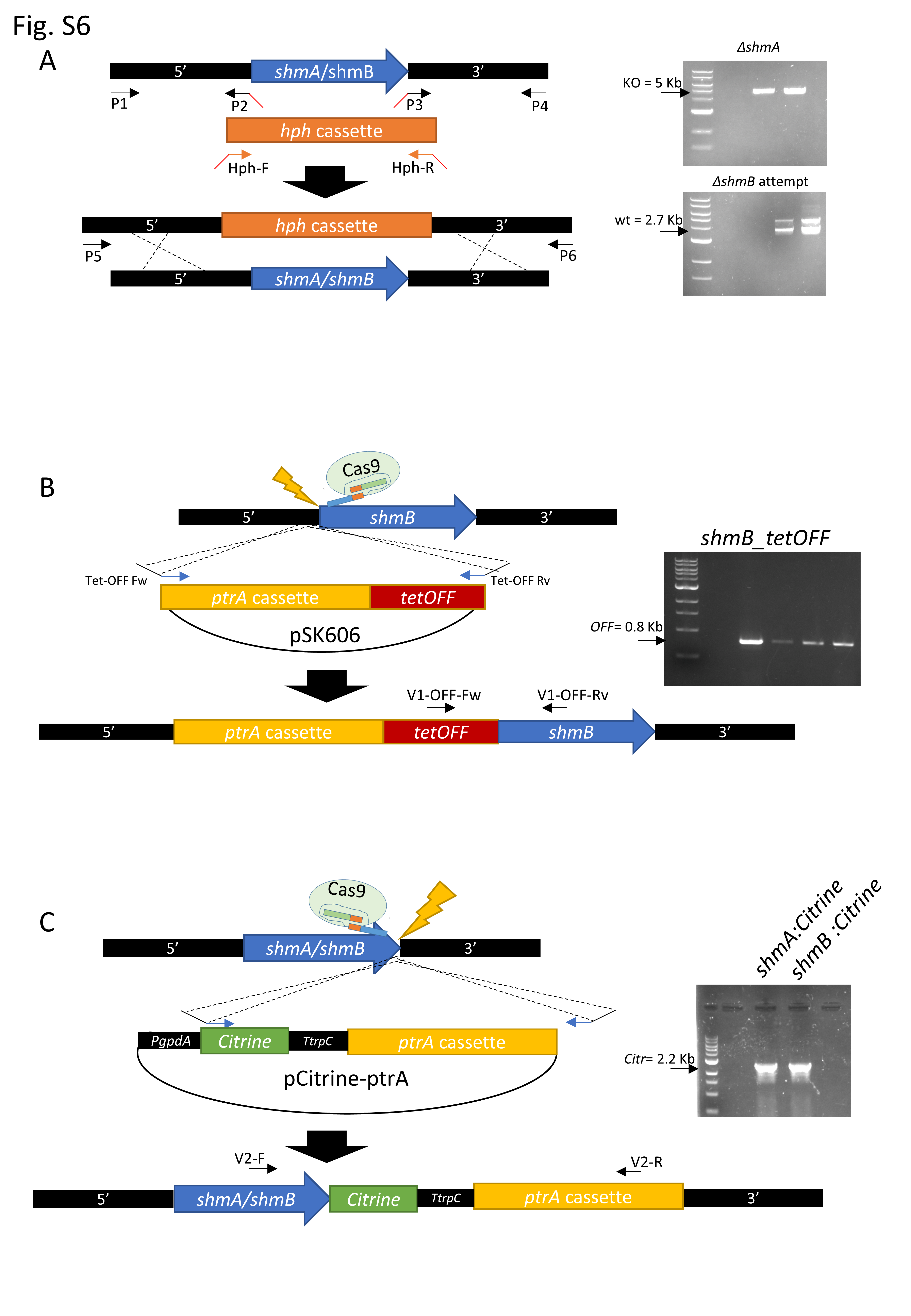

Supplement: Supplementary Figure 6.tif [file KVIR_A_2449075_SM1439.tif]
